# Detection of aphid infestation on faba bean (*Vicia faba* L.) by hyperspectral imaging and spectral information divergence methods

**DOI:** 10.1007/s41348-025-01100-6

**Published:** 2025-06-10

**Authors:** Ali Saeidan, John Caulfield, Jozsef Vuts, Ni Yang, Ian Fisk

**Affiliations:** 1 International Flavour Research Centre, Division of Food, Nutrition and Dietetics, University of Nottingham, Sutton Bonington Campus, Loughborough, LE12 5RD UK; 2 Biological Chemistry Department, Rothamsted Research, Harpenden, Hertfordshire AL5 2JQ UK; 3 Department of Bio Interactions and Crop Protection, Rothamsted Research, Harpenden, AL52JQ UK; 4 International Flavour Research Centre (Adelaide), School of Agriculture, Food and Wine and Waite Research Institute, The University of Adelaide, PMB 1, Glen Osmond, SA 5064 Australia

**Keywords:** Aphid detection, Hyperspectral imaging, Spectral information divergence, Machine learning

## Abstract

Aphids hide under leaves, reproduce rapidly, and require early detection to prevent crop damage, disease transmission, and ensure effective pest management. This study presents a novel approach for aphid detection by utilizing hyperspectral imaging, multivariate classification methods and spectral information divergence (SID) analyses. The hyperspectral images average spectrum (*n* = 336) showed significant differences between healthy and infested leaves. Time-series classification was performed over 14 days after infestation using four distinct machine learning algorithms. Early-stage infection detection may not relate to internal physiological alterations within the leaf but rather to the physical presence of the aphid behind the leaf, obstructing subtle physiological signatures. Implementation of spectral endmembers in the VIS–NIR reference spectrum led to the identification of an informative abundance SID map within the 710–825 nm range, useful for further classification. Machine learning classification resulted in support vector machines achieving 99.20 accuracy. Using random forest, twenty-two most important variables found effective in boosting classifier performance. The selected model also extended to real-world scenarios by testing progressing infestation patterns over 14 days on independent data sets, confirming the system’s reliability. Signal normal variant pre-treatment with partial least squares regression was effective in the estimation of aphid populations, achieving a 0.81 coefficient of determination (*R*^2^) and a 10.29 root-mean-square error of prediction for test datasets. In conclusion, the proposed method was able to successfully detect aphid colony infestation, both earlier and in locations that are invisible during standard human inspection.

## Introduction

Faba bean (*Vicia faba*, Leguminosae.) is a widely cultivated winter-sown leguminous crop known for its nutritional value, including high levels of carbohydrates, protein, minerals, and bioactive compounds (Karkanis et al. 2018; Lizarazo et al. 2015). The global market for faba beans has been steadily growing, with a projected compound annual growth rate (CAGR) of 2.6%. China leads as the largest producer with 1.69 million ton annual production, followed by Ethiopia, Australia, and the UK (FAOSTAT 2022). However, the productivity and quality of faba beans are threatened by the infestation of black bean aphids (Peignier et al. 2023). These aphids cause direct damage to the plants by feeding on the phloem, resulting in impaired growth and reduced yield (Shannag and Ababneh 2007). Furthermore, they act as vectors for plant viruses such as bean leaf roll virus (BLRV), alfalfa mosaic virus (AMV), and bean yellow mosaic virus (BYMV), leading to additional indirect damage (Abdelkhalek et al. 2023; El Amri 2001; Neeraj et al. 1999).

The rapid escalation of infestations, causing substantial damage to plants, is facilitated by the asexual reproduction and viviparity of aphids. Their short generation times and high reproduction rates contribute to the swift expansion of infestations, making even minor initial occurrences impactful on plant health (El Amri 2001). Aphids extract sap by suction and excrete honeydew on leaves, creating a favourable environment for fungal growth, which obstructs the absorption of specific wavelengths in the electromagnetic spectrum (550–650 nm) used for chlorophyll production. Visible effects of the pest on the crops occur when the infestation exceeds the economic threshold. In this study we used 50 aphid/leaf/plant as reference (Hernández et al. 2021). However, according to different sources including the Government of Saskatchewan (2025), economic thresholds can vary based on local conditions, crop varieties, and market factors. Therefore, consulting regional agricultural extensions or local experts is recommended for the most accurate and relevant information. 

Accurate monitoring and control of aphid populations are crucial for effective crop protection. Traditional methods, such as visual inspections and trapping, are time-consuming and fail to provide precise information on spatial and temporal aphid distribution, resulting in suboptimal pest management practices. Excessive pesticide applications lead to environmental hazards, negative impacts on beneficial insects, health risks, and insect resistance. To overcome these challenges, innovative technologies are needed for early pest detection before reaching economic thresholds (Ding and Taylor 2016; Ragsdale et al. 2007; van der Werf 1996). Hyperspectral imaging, particularly utilizing changes in plant reflectance caused by biotic/abiotic stressors, offers a promising solution. This non-destructive and rapid analysis technique enables accurate quantification of plant stress at fine resolutions, enhancing preventive measures. However, effective wavelength selection is needed to optimize the use of hyperspectral data and reduce computational complexity due to its high dimensionality and collinearity. Hyperspectral imaging has demonstrated success in various plant science applications, such as evaluating plant health parameters (Xiaobo et al. 2011; Zhang et al. 2015), detecting and monitoring plant diseases and pests (Baranowski et al. 2015; Kong et al. 2014; Lee 2015; Liu et al. 2016; Mahlein et al. 2010, 2012; Ochoa et al. 2016; Rajendran et al. 2016; Rumpf et al. 2010; Yeh et al. 2016; Zhu et al. 2016, 2017), and segmenting infested areas (Kumar et al. 2010; Varpe et al. 2015). 

Researchers have explored various hyperspectral techniques for detecting and quantifying aphid infestations. Machine learning models, such as one-class support vector machine and Laplacian of Gaussians blob detection, achieved exceptional validation scores in accurately identifying and quantifying aphids on leaves (Peignier et al. 2023). Near-infrared spectroscopy (NIR) and electronic nose (e-nose) coupled with artificial neural network (ANN) models have been utilized for classification and regression tasks, showing high accuracy in detecting infestation levels and predicting insect numbers (Fuentes et al. 2021). Specific narrow-band near-infrared wavelengths have been associated with aphid abundance in soybean (Alves et al. 2019), while wavelengths 788.17 nm, 965.14 nm, and 850 nm for detecting infestation have been identified in sorghum and cotton leaves (Hernández et al. 2021). Most of the previous studies have failed to consider the location of aphids (below leaf) as a game changing factor and their potential influence on leaf reflectance. So, they focus on imaging above leaf/step aphids, which are often only present during later stages of infestation.

Implementing routine field surveys for monitoring aphid infestations is often labour-intensive and may not precisely capture the spatial and temporal dispersion of pests. Consequently, adopting an automated, non-destructive methodology to detect and assess aphid infestations would yield advantages for enhanced precision and targeted treatments. While prior research has attempted to tackle this issue, it has been limited to the detection of aphids on the leaf surface (Peignier et al. 2023). However, aphid colonies predominantly accumulate beneath the leaves, making them invisible for human eyes, so addressing this specific concern is crucial in pest management. In this investigation, a machine learning model is introduced to identify and quantify aphids, in a more ecologically valid environment than previous studies (below leaf) through the application of multivariate classification methods and the spectral information divergence approach.

## Material and methods

### Sample preparation

#### Faba bean samples

Faba bean plants were cultivated in greenhouses at the Sutton Bonington campus, University of Nottingham, UK, using organic seeds provided by Rothamsted Research Harpenden, UK. Germination took place in a commercially available peat substrate (Klasmann low nutrient compost). Throughout the cultivation process, from the seedling stage onward, all plants received consistent light intensities and were grown in the same medium. Precise environmental control was ensured, maintaining the culture environment at an average temperature of 18.5 °C, with a maximum temperature of 20 °C and a minimum of 17 °C. The average relative humidity during cultivation was calculated to be 44.475%, with a recorded range between 32 and 56%. For this study, three-week-old plants (Ritchie et al. 1986) were employed. Each group of infested potted plants was confined within an insect rearing tent (50 × 25 × 25 cm), encompassed by 100-μm nylon mesh on all six sides. The entire plant setup was housed in a growth room following a 12-h daylight and 12-h night cycle.

#### Aphid infestation

**Fig. 1:**
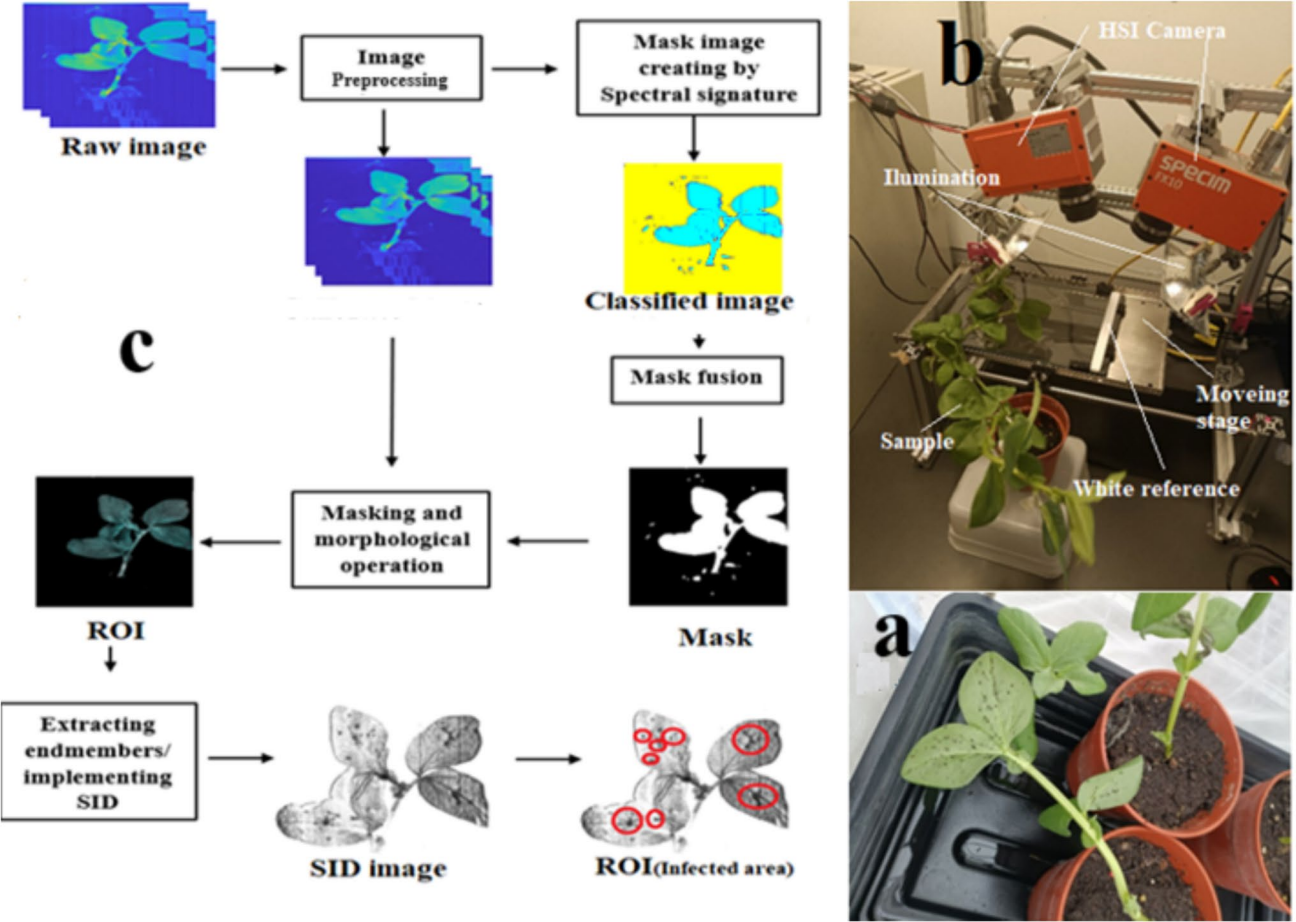
Image pre-processing pipeline (**a**), configuration of hyperspectral experimental setup (**b**), Aphid colony developed on faba leaf (**c**)

*Aphis fabae* Scopoli (Hemiptera: Aphididae), commonly known as black bean aphids, were sourced from Rothamsted Research, Harpenden, UK. The initial aphid colony was allowed to proliferate, resulting in an augmented population within a rearing tent housing faba plants. Subsequently, for each leaf in the infested group, five adult *A. fabae* individuals were randomly selected from the colony and introduced to the experimental plants, positioned beneath the leaves (Fig. 1a). A meticulous transfer of aphids into the faba plants was conducted using a fine natural bristle brush, and to prevent aphid escape, clip cages were promptly deployed on the first day of infestation.

#### Hyperspectral image acquisition

Time series hyperspectral imaging data were collected from both aphid-infested and healthy leaves over a fourteen-day time courses, covering pre-infestation as baseline (day 0) and post-infestation time points daily from day 1 to day 14, with two biological replicates for each time point. The imaging process occurred near a climate-controlled room where the experimental units were housed, with each cage of plants transferred one at a time to the imaging room, ensuring efficient imaging within 1–2 min per plant. The push broom hyperspectral imaging system (Fig. 1b) consisted of an HSI camera, Specim FX10, equipped with halogen lighting, mounted on a Specim LabScanner sized 40 × 20 cm (Specim, Spectral Imaging, Ltd., Oulu, Finland). Working within the visible near-infrared (VNIR) spectral range of 380–1000 nm, the operated camera with a mean spectral resolution of 5.5 nm (224 bands) and line comprising 1024 spatial pixels, could acquire spectral data at a maximum frame rate of 327 frames per second (fps) in full-frame mode. The hyperspectral measurements were conducted in a dark room, illuminated solely by LabScanner halogen lamps, and one scans were acquired for each hyperspectral dataset, which was saved in raw format for subsequent processing. Reflectance calibration was performed for each scan, utilizing white and dark images to obtain corrected images through a specific equation (Saeidan et al. 2021). After horizontal positioning and the leaf reflectance image in each spectral band was computed for all combinations of faba plant and time point, forming the basis for subsequent statistical analyses. The dataset comprised fourteen time points (ranging from 1 to 14 days), two plant treatments, two replications and six hyperspectral images for each treatment, resulting in a total of 336 reflectance image (observations) that were included in the statistical analyses.

### Data analyses

#### Pixel selection and pre-treatments

**Fig. 2:**
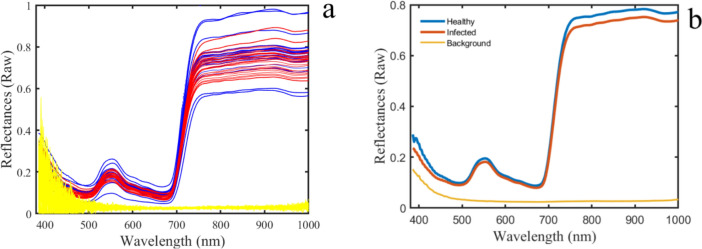
Near-infrared curves illustrating the reflectance values across a wavelength range of 380–1000 nm for the raw spectra (**a**) and the average spectra for background, healthy and infested plants observed over a period of 14 days (**b**)

Data analysis was performed using Matlab software (MATLAB 2018). As shown in Fig. 1c, to distinguish between the background and plant areas in hyperspectral images, a simple basic classifier (SVM) was utilized. The dataset, gathered manually, consisted of two groups of spectral signatures representing plant and background, with approximately 300 pixels in each category. Because of the significant contrast between the background signature and the plant material (see Fig. 2a), segmenting the hyperspectral image into two groups was a straightforward task. To eliminate pixels with mixed spectral characteristics, the resulting mask subjected to morphological operation called “erosion” using a (5 × 5) matrix as the structuring element (Barreto et al. 2020; Hirata and Papakostas 2021). Consequently, the resulting mask effectively limited the region of interest (ROI) only for plant area. ROIs of healthy and infested plants were mapped onto original spectral images to obtain full spectra in the 380–1000 nm range. The samples were processed under consistent conditions, resulting in a spectral dataset. An average value for each plant was employed for classification, resulting in 336 samples, evenly distributed across infested and healthy faba bean groups. The dataset was divided into training and test sets, with 60% (200 spectra) used for training classification models, and the remaining 40% (136 spectra) employed as the test set. To ensure comparability, the spectral intensity in each waveband was normalized (standard normal variant) using the average and standard deviation intensity values from the entire spectrum (Zhang et al. 2016). Second part of the data processing continued by extracting a reference spectrum based on endmember values and comparing all spectra with this reference to obtain binary masks for infested plant images. End-members are fundamental spectral signatures in hyperspectral data that represent unique materials. They facilitate the decomposition of image pixels into fractional material abundances. These spectra are typically considered pure pixels and can be detected using geometric and convex analysis methods (Winter 1999). To choose only those pixels associated with pure aphid signature the Spectral Information Divergence (SID) algorithm was utilized. In the SID, a reference spectrum is a known spectral signature used as a comparison baseline to measure the similarity between different spectral signatures in hyperspectral data. The SID algorithm then computes the spectral divergence between the reference spectrum and each pixel spectrum in the image to assess their similarity (Yousefi et al. 2016). Process continued by manual selection of aphid-accumulated areas for each sample using roipoly command from the Matlab image processing toolbox. Relevant spectra for the infested dataset were then extracted from pixels.

#### Spectral similarity measures

In this study, we address the detection of aphids in hyperspectral images using spectral similarity measures. Initially, the task was approached as a binary classification problem; however, due to unknown nature of infestation pattern distribution on leaves, negative class examples were not representative of pure aphid infestation (Peignier et al. 2023). To overcome this limitation, the SID method was adopted, enabling the evaluation of aphid infestation discriminatory ability. This method calculates the distance between the probability distributions produced by two spectral signatures and a lower divergence value indicates higher similarity between the vectors. The SID is mathematically defined using relative entropy or Kullback–Leibler information divergence.

Considering hyperspectral pixel (vector) $x={\left({x}_{1},{x}_{2}, \dots ,{x}_{L} \right)}^{T}$ representing a spectral vector acquired at various wavelengths ${{\varvec{\lambda}}}_{{\varvec{l}}}$**,** a probability vector $p={\left({p}_{1},{p}_{2}, \dots ,{p}_{L} \right)}^{T}$**,** characterizes the statistics of the pixel x (Eq. 1), Using (1) entropy of each hyperspectral image pixel x can be defined as Eq. 2. Additionally, considering another pixel $y={\left({y}_{1},{py}_{2}, \dots ,{y}_{L} \right)}^{T}$ with its corresponding probability vector $q={\left({q}_{1},{q}_{2}, \dots ,{q}_{L} \right)}^{T}$ the self-information for the l-th band of pixels x and y is introduced using information theory (Eq. 3). Then using (3), the relative entropy of y with respect to x can be defined by Eq. 4.

1 $$p\left( {\left\{ {\lambda_{j} } \right\}} \right) = p_{j} = {\raise0.7ex\hbox{${x_{j} }$} \!\mathord{\left/ {\vphantom {{x_{j} } {\mathop \sum \nolimits_{l = 1}^{L} x_{l} }}}\right.\kern-0pt} \!\lower0.7ex\hbox{${\mathop \sum \nolimits_{l = 1}^{L} x_{l} }$}}$$

2 $$H\left( x \right) = - \mathop \sum \limits_{l = 1}^{L} p_{l} \log p_{l}$$

3 $$I_{l} \left( x \right) = - \log p_{l} and I_{l} \left( y \right) = - \log q_{l}$$

4 $$D\left( {x ||y} \right) = \mathop \sum \limits_{i = 1}^{L} p_{l} D_{l} \left( {x ||y} \right) = \mathop \sum \limits_{i = 1}^{L} p_{l} \left( {I_{l} \left( y \right) - I_{l} \left( x \right)} \right) = \mathop \sum \limits_{i = 1}^{L} p_{l} \log \left( { \frac{{p_{l} }}{{q_{l} }}} \right)$$

Building upon this, the Kullback–Leibler information measure, quantifying the difference between the probability distributions of x and y, is defined as the relative entropy D (x ‖y). The assessment of spectral similarity between pixels x and y involves the proposition of the SID as a symmetric hyperspectral measure, which is computed as:

5 $$SID\left( {x ||y} \right) = D\left( {x ||y} \right) + D\left( {y||x} \right)$$

By incorporating relative entropy, SID offers a novel perspective on spectral similarity, enabling a more informative characterization of each pixel’s spectral information (Chang 2000).

#### Feature selection

Principal component analysis (PCA) is a widely used dimensionality reduction (DR) technology and implemented in this study to reduce dimensionality of data and eliminate multi-collinearity while retaining maximum variance (Hasan and Abdulazeez 2021; Omuya et al. 2021). In this study, we implemented PCA on raw data and incorporated first three principal components as an input for one of the classification treatments. Also, in later phase of research and to feed relevant feature to each classifier, twenty-two distinctive wavelengths (features) on spectrums of healthy and pure infested area on leaves were selected by random forest (RF) method. RF, recognized as one of the most prevalent ensembles learning techniques for feature selection tasks, entails the creation of numerous decision trees that are uncorrelated. Specifically, RF effectively utilizes the "Wrapper" method to generate scores indicating the importance of variables (Iranzad and Liu 2024). In this research, we developed a RF algorithm in Matlab environment utilizing Bagging method and the number of decision trees set to 200.

#### Classification modelling techniques and performance

In this research, a diverse set of machine learning techniques, including support vector machine (SVM), linear discriminant analysis (LDA), K-nearest neighbourhood (KNN), and artificial neural network (ANN), were applied. The results of using these four approaches were compared on the accuracy and errors for early detection of aphid in faba bean. The fitting and validation of these models were conducted using the Matlab statistical software (Matlab 2018). The dataset used for modelling comprised a total of 336 samples, with 168 samples representing healthy plants and the remaining 168 samples for inoculated plants. As part of the supervised classification approaches, LDA was employed. This method aims to maximize data variance and model differences between various data classes. By identifying linear combinations of independent variables, LDA effectively separates different classes of objects or events, projecting the multidimensional feature space into a lower-dimensional space where the ratio of between-class scatter to within-class scatter is optimized. SVM, based on discovering the most suitable line or decision boundary that proficiently segregates data points associated with distinct classes, was utilized to classify samples into two groups by constructing an optimal hyperplane. This approach is well-suited for small sample learning problems, as well as handling nonlinear and high-dimensional datasets (Kok et al. 2021). A radial basis function (RBF) was selected as the kernel function, a choice that has demonstrated successful outcomes in numerous previous studies (Peignier et al. 2023). The study employed the KNN algorithm as another classifier. This nonparametric machine learning technique identifies the k-nearest neighbours in the training data that are closest to the test value and calculates the distance between the test value and each of those neighbours. Additionally, an ANN with three layer and “Relu” activation function was implemented as classifier. For the evaluation of binary classification performance in the context of imbalanced conditions, we adopted standard evaluation measures, specifically the F1 score, precision, and the area under the curve (AUC). To compute these evaluation measures for different validation sets, a tenfold was employed during cross-validation procedure. This method ensures robustness and consistency in the assessment process, particularly when dealing with imbalanced datasets.

## Results

### Classification results when incorporating spectrums of entire leaf

After calibration, the resulted unprocessed (raw reflectance) spectra of individual plant samples, as well as their average, are illustrated in Fig. 2. The average spectrum reveals subtle distinctions between healthy and infested samples, predominantly within the spectral range of 725–1000 nm. Specifically, for healthy leaves, the reflectance between 725 and 1000 nm exhibits a notably higher level compared to other parts of the spectral curve. This is in accordance with Alves et al. (2019), which claimed, reflectance in the near-infrared spectral range were negatively associated with cumulative aphis days (CAD), but those in the visible spectral range were not associated with CAD.

This study used four distinct machine learning algorithms to classify samples into "Healthy" and "Infested" categories using both raw and pre-processed spectral data. The pre-processing involved the standard normal variate (SNV) method and feature selection via principal component analysis (PCA). Table 1 presents the model performances based on various parameter combinations and their corresponding optimal settings. The mean overall accuracy was computed across 50 iterations of tenfold cross-validation for each parameter combination, along with average values for area under the curve (AUC), precision, F1 scores, and training and test accuracies during cross-validation. The findings highlight that the ANN and SVM models outperformed the other two algorithms in terms of overall accuracy, indicating their robustness. The mean overall test accuracy across different algorithm ranged from 0.65 to 0.95, accompanied by corresponding AUC values spanning from 0.62 to 0.93. F1 and precision scores ranged from 0.54 to 1.00 and 0.63 to 1.00, respectively. Among the algorithms, SVM and ANN models exhibited superior performance, while KNN model also delivered good results. In contrast, the LDA model demonstrated comparatively less favourable outcome.

**Table 1:** The summary of classification measures, including accuracy, AUC (area under the curve), F1 scores, and precision scores, achieved by four different classification methods: linear discriminant analysis (LDA), support vector machine (SVM), K-nearest neighbour (KNN), and artificial neural network (ANN)

	Pre-treatment/Feature selection	CV-validation	Test	AUC		F1		Precision		
LDA					**A**		**B**		**A**	**B**
	Raw	0.78	0.70	0.79	0.70		0.63		0.68	0.77
	SNV	0.83	0.90	0.91	0.79		0.72		0.76	0.86
	PCA(3pc)	0.70	0.90	0.79	0.67		0.54		0.63	0.79
ANN	Raw	0.80	0.70	0.85	0.76		0.78		0.80	1.00
	SNV	0.81	0.80	0.83	0.78		0.75		0.80	1.00
	PCA(3pc)	0.95	0.95	0.69	0.67		0.55		0.70	1.00
KNN	Raw	0.76	0.80	0.84	0.69		0.62		0.64	0.66
	SNV	0.78	0.70	0.81	0.74		0.68		0.76	1.00
	PCA(3pc)	0.73	0.90	0.80	0.72		0.59		0.70	0.95
SVM	Raw	0.83	0.60	0.89	0.86		0.83		0.92	1.00
	SNV	**0.95**	**0.90**	**0.94**	**0.93**		**0.86**		**0.88**	**0.95**
	PCA(3pc)	0.78	0.85	0.81	0.76		0.66		0.76	1.00

In this table, A and B refer to two different classes, respectively, for healthy sample and infested ones

Bold values demonstrate the best performance model

**Fig. 3:**
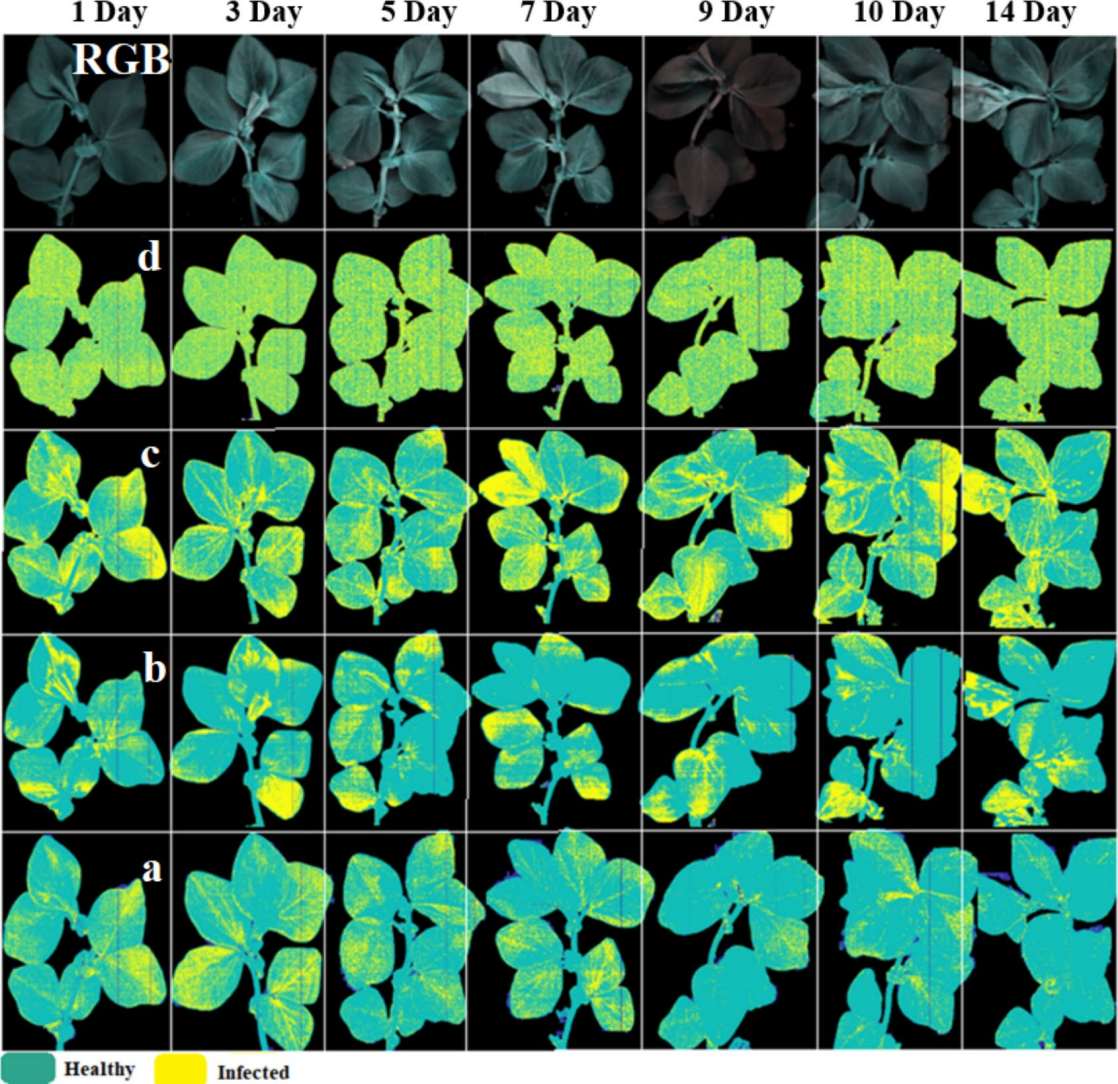
Pixel-wise classification of spectral images of healthy plants over day 1 to day 14. Probability response to classify a pixel adjacent to a healthy plant using various models: **a** SVM model, **b** KNN model, **c** ANN model, and **d** LDA model. All models were trained with signals pre-processed using SNV

**Fig. 4:**
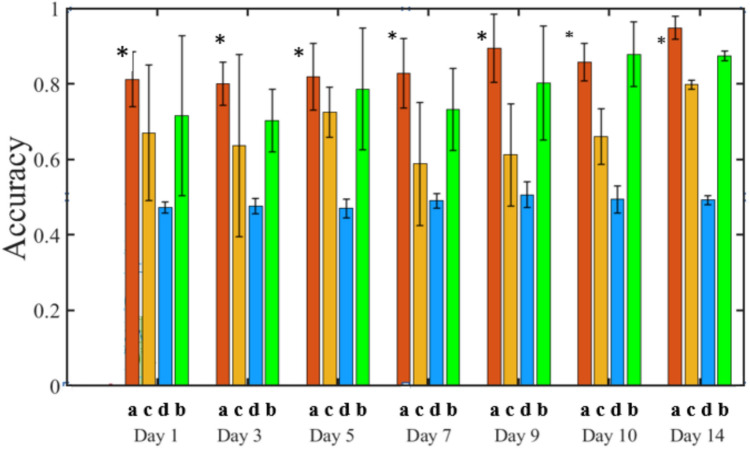
Accuracy of best models. Performances are exposed for each subset/approach over 14 days and based on counting true-positive and false-negative pixels. (*) Asterisk marks indicate the best machine learning model with the highest average accuracy value

Considering different spectral datasets, models using SNV-processed spectra performed well for both SVM and ANN, while PCA-treated spectra yielded better results for ANN. Overall, SVM with SNV-pre-processed spectra as input variables demonstrated the best performance during tenfold cross-validation, achieving a mean accuracy of 0.95 and an AUC of 0.94. Notably, these models trained efficiently, completing the process within seconds. As shown in Fig. 3, the performance of four classifiers was evaluated over 14 days on an independent set of healthy samples. The green colour represents healthy pixels, while yellow pixels indicate misclassified infested ones. Ideally, no signs of infestation should appear in healthy samples; however, in this figure, nearly all classifiers fail to classify all pixels as healthy. Comparing the classifiers, the bottom row, representing the SVM classifier (labelled “a”), demonstrates the highest accuracy in identifying green pixels as healthy. The KNN classifier ranks second in accuracy, followed by ANN. The LDA classifier, shown in the second row, exhibits very poor classification performance, misclassifying nearly half of the healthy pixels as infested (Fig. 4).

The representation of infested samples over 14 days using the SVM_a classifier, trained on data collected from the entire leaf including both aphid-accumulated areas and un-accumulated regions is provided in Fig. 8. While we will explore this figure in detail in subsequent sections, it is important to note that, contrary to our initial expectations, indications of infestation were evident from the first day. One possible explanation for this observation is the accumulation of aphids beneath the leaf, which may interfere with light reflection. We hypothesize that a portion of NIR light penetrates the leaf, interacts with the aphid’s body, and is subsequently reflected towards the camera (Peignier et al. 2023).

However, the classification of certain infested pixels as yellow may be attributed to specific physical alterations occurring within the leaf (Barreto et al. 2020; Luo et al. 2011). Figure 4 illustrates that after fourteen days of aphid infestation, the number of healthy pixels within the healthy group gradually approaches 1.00, aligning with expectations.

### Classification results when incorporating aphid-accumulated area on leaf

To disentangle spectral signatures resulting from physiological changes and those associated with aphid bodies, pinpointing the exact location of aphids is crucial. This enables the creation of a region of interest (ROI) limited to the aphid-affected area for data extraction and processing (Peignier et al. 2023). In the ensuing step, meticulous feature selection was employed to identify which NIR wavelengths are associated with the aphid’s body beneath the leaf.

**Fig. 5:**
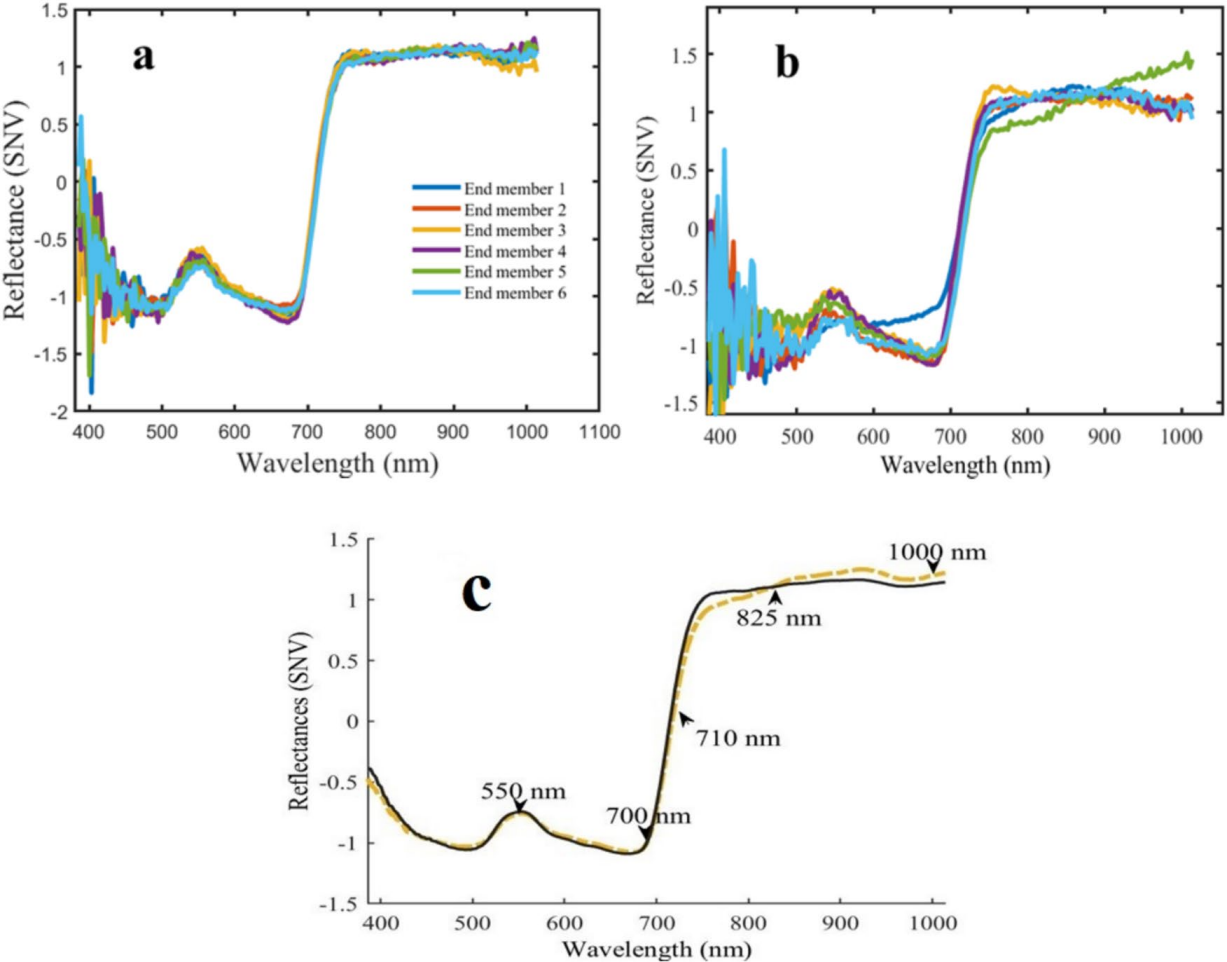
Six endmembers derived from SNV pre-treated **a** healthy leaves, **b** leaves with aphid and **c** the average spectra’s, calculated from healthy and infested endmembers. Three different regions including 550–700 nm, 710–825 nm, 825–1000 nm on average spectrums were used as input for SID algorithm

The Nfinder algorithm (Winter 1999) was employed on the hyperspectral image dataset, resulting in six distinct endmembers. These endmembers were used to unmix the original hyperspectral images through a straightforward linear unmixing process. Notably, all endmembers within the healthy group (Fig. 5a) during Day 10 exhibited nearly identical spectral signatures, whereas plants affected by aphids exhibit notable discrepancies in certain spectral regions (Fig. 5b). Specifically, the ranges of 600–700 nm (endmember 1) and 700–850 nm, as well as 850–1000 nm (endmember 5), are indicative of the aphid signature (Bajwa et al. 2017), a finding that aligns with the work of Peignier et al. (2023).

**Fig. 6:**
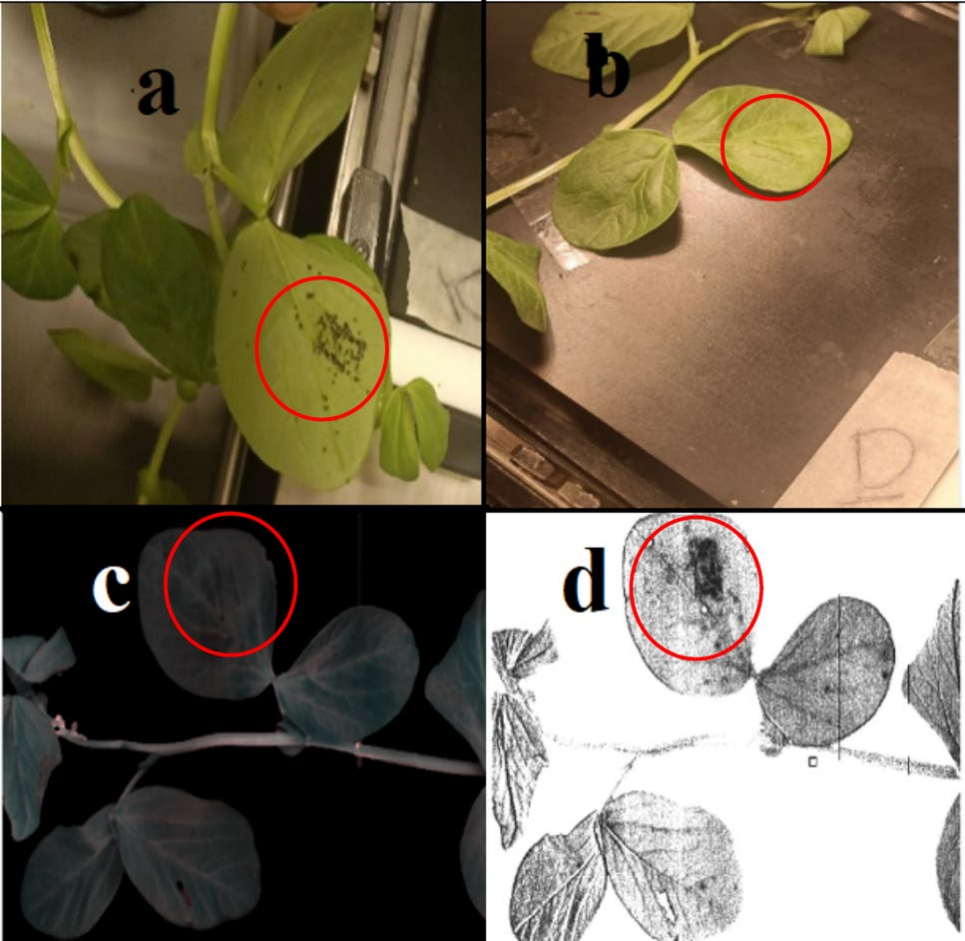
Demonstration of invisible aphid pattern beneath the leaf in the form of RGB image captured by handheld camera (**a**), (**b**) and FX 10 camera (**c**) and SID abundance map (**d**)

The study utilized the average endmember spectrum (Fig. 5c) from infested plants as a reference for the SID algorithm, enabling the identification of regions of interest (ROIs) and subsequently extract target spectrums. The SID-based approach was applied to hyperspectral imagery across three distinct spectral ranges, revealing scores within the 710–825 nm range effectively highlighting aphid accumulation beneath the leaves (Fig. 6d). It should be mentioned that the aphids positioned behind the leaves are completely invisible for human eyes (Fig. 6b, c).

**Fig. 7:**
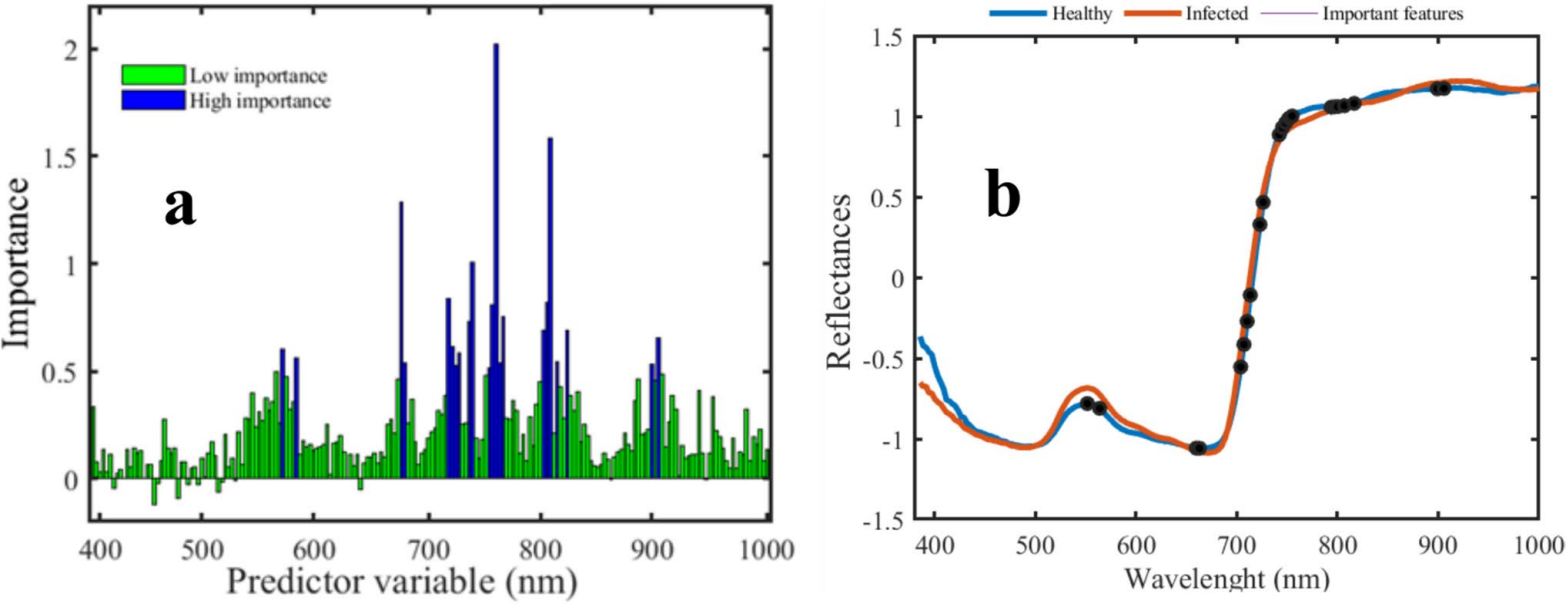
Feature ranking results based on variable importance parameter. Twenty-two wavelengths have high importance value greater than 0.5 shown in blue (**a**). Representation of average healthy sample and infested ones along with selected wavelengths (**b**)

These spectrums, extracted from pure infested area, were combined with previously collected uncontaminated healthy ones to create a comprehensive final data matrix. Then as shown in Fig. 7, the RF feature selection method was used to identify the most important wavelengths based on variable scores. Feature selection algorithm selects twenty-two wavelengths as important variables which fourteen of them (705 nm, 707 nm, 710 nm, 713 nm, 723 nm, 726 nm, 742 nm, 745 nm, 748 nm, 751 nm, 755 nm, 794 nm, 797 nm, 800 nm) distributed across 700–800 nm region. Four wavelengths fall within the visible range (550 nm, 564 nm, 660 nm, and 663 nm), while another four are identified between 800 and 900 nm.

Four distinct classification methods were applied to categorize these data into “healthy” and “infested” groups, and their performance metrics were evaluated using a tenfold cross-validation approach, as summarized in Table 2.

**Table 2:** Average tenfold cross-validation of accuracy, AUC, F1, and precision scores obtained by the four different classification models which used SNV pre-treated wavelengths and 22 selected features

	Pre- treatment	CV-validation	Test	AUC	F1 Precision			
					A	B	A	B
LDA	SNV(22 features)	98.80	98.60	1	0.98	0.98	0.98	0.94
SVM	SNV(22 features)	**99.00**	**99.20**	**1**	**0.98**	**0.98**	**0.98**	**0.93**
KNN	SNV(22 features)	95.90	97.10	0.99	0.95	0.97	0.98	0.95
ANN	SNV(22 features)	98.70	98.90	0.99	0.98	0.98	0.98	0.92

Bold values demonstrate the best performance model

**Fig. 8:**
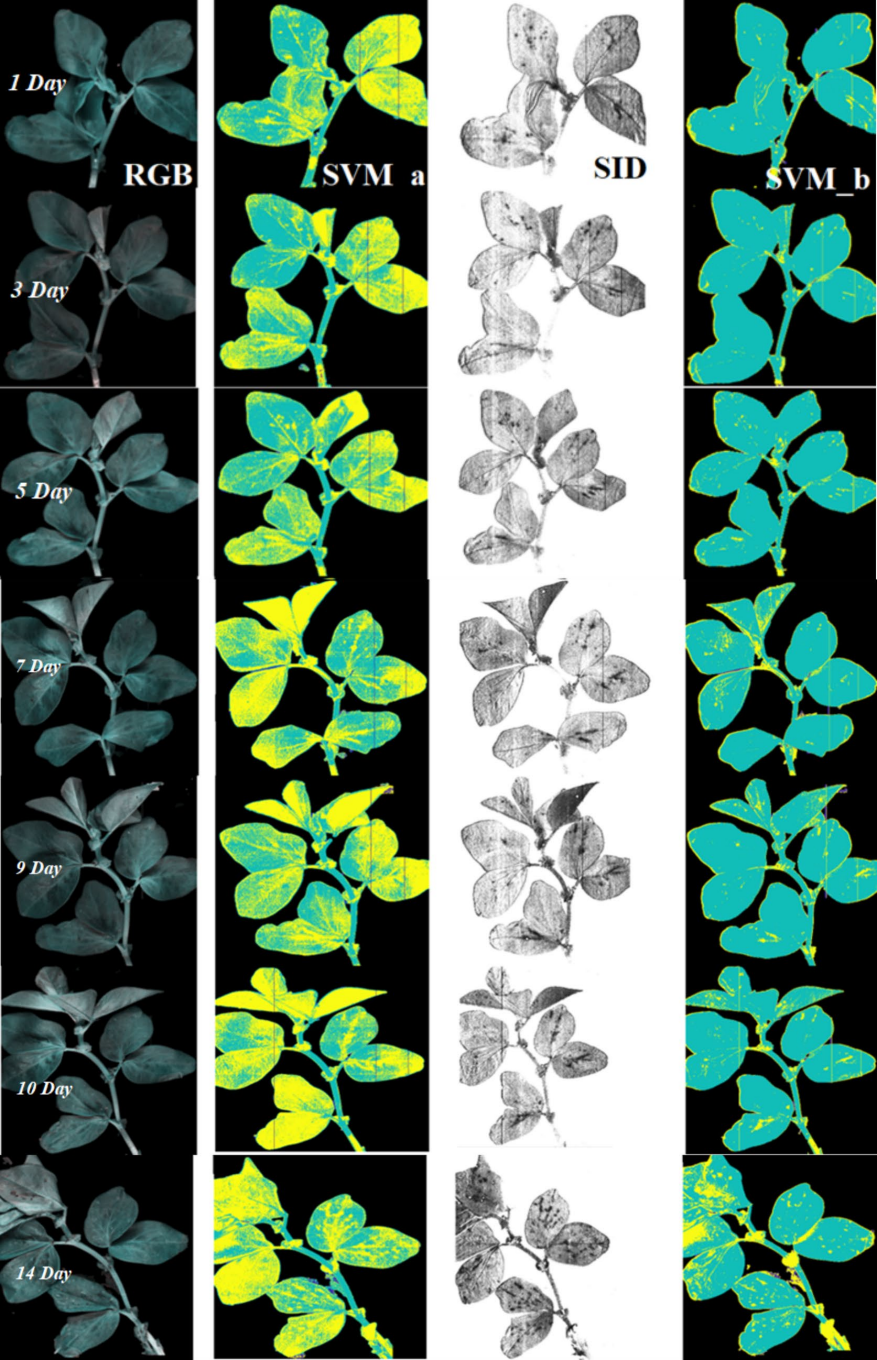
Classification performance on independent data set (*n* = 7 HIS images). Column (1) represents RGB image, column (2) shows SVM trained on all wavelengths, column (3) shows spectral information divergence (SID) abundance map and column (4) indicates SVM classification trained on 22 selected features)

The classification pipelines yielded similar and high-quality results. While two methods, KNN and ANN, exhibited lower precision scores, most methods delivered satisfactory scores. F1 scores were comparable across methods, except for the KNN scheme, which scored lower. The area under the curve (AUC) consistently demonstrated high-quality outcomes, with the SVM method surpassing the other methods in terms of accuracy and precision. To thoroughly assess the system under the challenging scenarios with the data originated from independent sets which was not included in training/testing process. The performance of the SVM classifier trained with the twenty-two most important variables was showcased through pixel-wise representation in Fig. 8. Over the 14-day period depicted in this figure, aphid infestation advanced in tandem with the rise in aphid population. In the first column, the RGB image of the faba plant revealed that the aphids beneath the leaves had become completely invisible to human eyes. The second column displayed the outcomes of SVM classification, trained with all features. As a reminder, we collected these data set from all surface area of infested plant regardless of those area which aphid accumulated.

Moving to the third column, an image was generated based on the SID score, clearly highlighting areas where aphids had aggregated beneath leaves. This image can be served as a ground-truth reference for the last column. In the last column, the results of SVM (SVM_b) classification, incorporating 22 selected features during training, were evaluated. The accurate classification performance was discernible by comparing the exact aphid locations in the previous column (SID image). However, it is important to acknowledge that while SVM_b was effective in distinguishing between aphid accumulation areas and other leaf regions, it was not without imperfections.

### Estimating aphid papulation on leaf

In addition, the spectrums collected manually only from aphid accumulation area on leaf were employed to estimate the aphid population beneath the leaves. To achieve this, partial least square (PLS) regression models in conjunction with various data pre-processing methods including standard normal variate (SNV), Log (1/R), first and second derivatives were used. It is worth noting that these pre-processing approaches had only a slight impact on the final prediction error, as summarized in Table 3. Among the pre-processing methods, the SNV treatment demonstrated the most favourable calibration performance for the samples, resulting in the highest calibration R-squared (*R*^2^) of 0.88. The cross-validation *R*^2^ (*R*^2^cv) values were 0.57, and the test *R*^2^ (R^2^prediction) reached 0.81.

**Table 3:** Performance of the PLS regression model for predicting aphid number by selected SVM_b classifier

Pre-processing method	LV	${R}^{2} Cal$	RMSEC	${R}^{2} CV$	RMSECV	${R}^{2} Prediction$	RMSEP
Raw	27	0.86	8.25	0.62	15.22	0.61	14.56
SNV	**19**	**0.88**	**7.96**	**0.57**	**16.93**	**0.81**	**10.29**
Log(1/R)	46	0.76	12.25	0.63	17.92	0.61	13.71
First derivative	26	0.83	9.94	0.61	17.51	0.80	11.29
Second derivative	18	0.83	9.61	0.42	19.47	0.75	11.37

Bold values demonstrate the best performance model

**Fig. 9:**
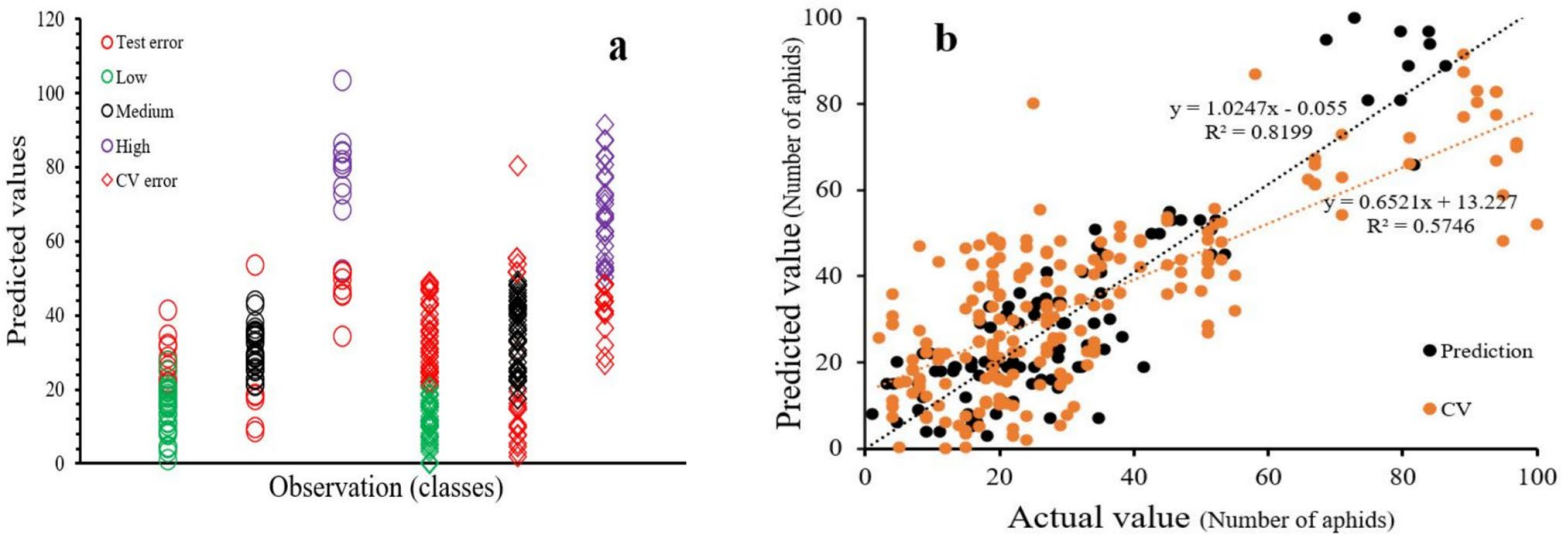
Comparison between predicted and reference values of aphid numbers. **a** categorizes the aphids into three different infection severity classes (low, medium, high) shows as green, black, and purple circle. Test error and cross-validation error were illustrated by red circle and red diamond. **b** Overall regression model statistics for predicted versus measured values of aphid numbers (black dot for prediction and orange dot for cross-validation). The predictions are generated using the SVM model with data spanning days 1 to 14

When modelling data from the “plants with aphids” reference dataset, the application of spectral pre-processing had a minimal effect on the prediction models. Calibration models exhibited *R*^2^ values ranging from 0.76 (using Log(1/R)) to 0.88 (with SNV). However, more significant differences emerged when assessing cross-validation and external validation (prediction) datasets. In these cases, the use of second derivative and raw spectra resulted in less favourable *R*^2^ values and higher prediction errors. As anticipated, first derivatives yielded similar prediction performance (R^2^cal = 0.83), given their function of noise reduction. SNV also delivered strong results, as it effectively mitigated light scattering effects and consequently led to a slightly lower prediction error. Nonetheless, the SNV treatment proved to be the most effective, as it consistently yielded the best performance with both calibration and prediction *R*^2^ values ranging from 0.88 to 0.81. In Fig. 9a, we present the predicted number of aphids in faba plants based on the best-performing prediction models. Furthermore, this figure provides a detailed account of the Model SVM-SNV, indicating that it exhibited categorized of aphid in three different (Low x < 20, Medium 20 < x < 50, High > 50) group based on infestation severity. Notably, the highest number of outliers was observed for class “Low” (12 out of 30) due to probable confusion in data extraction from ROI. Additionally, this model displayed relatively small rate of misclassification (6 out of 26) for class “Medium” and (5 out of 12) for class “High”.

Figure 9b shows relationships between observed and predicted number of aphids for the cross-validation and test set. The predictions are generated using the most effective model (SVM_b pre-treated with SNV) models with data spanning Days 1 to 14The R_square value for test set (prediction) is 0.81 which indicate goodness of fit. The variance of the error is almost constant across various levels of dependent variable, so it is statistically significant (Caporaso et al. 2022).

## Discussion

This study provides valuable insights into the detection and classification of aphid infestations using hyperspectral imaging. The reflectance spectra revealed subtle but significant variations between healthy and infested samples, particularly within the 750–900 nm spectral range. A possible explanation for this reduction in reflectance in the visible and NIR regions is the depletion of photosynthetic pigments, such as chlorophyll, due to aphid feeding, which negatively impacts plant photosynthesis (Riedell and Blackmer 1999; Morgham et al. 1994; Malthus and Madeira 1993). Notably, aphid infestations were detectable from the first day, suggesting that the interaction of NIR light with aphid bodies beneath the leaf surface allows for early detection (Peignier et al. 2023).

The study also highlighted differences in classifier performance. Parametric methods like LDA exhibited stable but less adaptive accuracy, while nonparametric classifiers such as SVM, ANN, and KNN demonstrated superior adaptability by dynamically adjusting decision boundaries as infestation patterns evolved (Graf et al. 2024). This adaptability made SVM, ANN, and KNN more effective for hyperspectral classification tasks.

Spectral analysis of aphid-infested plants further identified key variations in reflectance across 600–1000 nm, with pronounced differences in regions such as 550–700 nm, 710–825 nm, and 825–900 nm. Among these, the 710–825 nm range provided the most informative results when analysed using the SID algorithm. The resulting abundance map effectively highlighted aphid-affected regions, serving as a reliable region of interest (ROI) for classification (Pathak et al. 2023; Yousefi et al. 2016). These findings align with previous studies that associate aphid-induced changes in plant reflectance with modifications in leaf ultrastructure, secondary metabolite production, and physical damage caused by aphid feeding (Marston et al. 2020; Li et al. 2008; Kumar et al. 2013).

Additionally, aphids exhibit distinct reflectance properties, particularly in the NIR spectrum, which may be attributed to structural factors such as cuticle organization and pigmentation. These properties may serve as an adaptive mechanism, influencing their visibility under hyperspectral imaging (Mielewczik et al. 2012; Jacquemoud and Ustin 2019).

Despite relatively high root-mean-square error (RMSE) values in aphid population estimation, the classification models effectively categorized infestation levels. Given the variability in aphid populations, these prediction errors are acceptable for practical applications such as infestation screening and severity classification. These findings suggest that hyperspectral imaging, combined with machine learning, is a promising tool for precision agriculture, enabling early detection and improved monitoring of aphid infestations.

## Conclusion

In conclusion, this study demonstrates the effectiveness of hyperspectral imaging, coupled with advanced machine learning techniques, for the detection and monitoring of aphid infestations in plants. The results show that spectral signatures, particularly in the near-infrared range, can be leveraged to distinguish between healthy and infested leaves, with SVM and ANN classifiers providing the most robust performance. The incorporation of aphid accumulation areas through ROI-based spectral analysis (SID) significantly enhanced classification accuracy, while feature selection methods such as RF further improved model performance. Additionally, the use of PLS regression for aphid population estimation highlights the potential of hyperspectral imaging as a tool for both pest detection and quantitative analysis in agricultural settings. Although some misclassification occurred, especially in the low infestation category, the overall results suggest that hyperspectral imaging, when combined with appropriate data processing and machine learning techniques, can offer a reliable and efficient approach for pest management and monitoring in agricultural practices. Future studies could refine these models to reduce prediction errors and expand their applicability to other pest species and plant types. 
